# Quantitative Proteomics After Spinal Cord Injury (SCI) in a Regenerative and a Nonregenerative Stage in the Frog Xenopus laevis*[Fn FN2]

**DOI:** 10.1074/mcp.RA117.000215

**Published:** 2018-01-22

**Authors:** Dasfne Lee-Liu, Liangliang Sun, Norman J. Dovichi, Juan Larraín

**Affiliations:** From the ‡Center for Aging and Regeneration, Millennium Nucleus in Regenerative Biology, Faculty of Biological Sciences, P. Universidad Católica de Chile, Alameda 340, Santiago, Chile ;; §Department of Chemistry, Michigan State University, East Lansing, Michigan 48824;; ¶Department of Chemistry and Biochemistry, University of Notre Dame, Notre Dame, Indiana 46556

## Abstract

The capacity to regenerate the spinal cord after an injury is a coveted trait that only a limited group of nonmammalian organisms can achieve. In *Xenopus laevis*, this capacity is only present during larval or tadpole stages, but is absent during postmetamorphic frog stages. This provides an excellent model for comparative studies between a regenerative and a nonregenerative stage to identify the cellular and molecular mechanisms that explain this difference in regenerative potential. Here, we used iTRAQ chemistry to obtain a quantitative proteome of the spinal cord 1 day after a transection injury in regenerative and nonregenerative stage animals, and used sham operated animals as controls. We quantified a total of 6,384 proteins, with 172 showing significant differential expression in the regenerative stage and 240 in the nonregenerative stage, with an overlap of only 14 proteins. Functional enrichment analysis revealed that although the regenerative stage downregulated synapse/vesicle and mitochondrial proteins, the nonregenerative stage upregulated lipid metabolism proteins, and downregulated ribosomal and translation control proteins. Furthermore, STRING network analysis showed that proteins belonging to these groups are highly interconnected, providing interesting candidates for future functional studies. Data are available via ProteomeXchange with identifier PXD006993.

Spinal cord injury (SCI)^1^ in mammals, including humans, has irreversible consequences because of the low regenerative capacity of the mammalian central nervous system (CNS). After SCI, massive death of neurons and glia occurs, with the concomitant interruption of ascending and descending axonal tracts, causing loss of sensory and motor function among other consequences that severely affect the quality of life (1, 2).

Unlike mammals, amphibians and teleost fish possess a remarkable regenerative capacity that includes the CNS (3–5). Anurans or tailless amphibians, like the African clawed frog *Xenopus laevis*, display a high regenerative capacity during tadpole or larvae stages that is lost during metamorphosis, the thyroid hormone-dependent process during which tadpoles turn into froglets. Metamorphosis in *Xenopus laevis* starts with premetamorphosis (stages 46–54), when limb buds appear in tadpoles; followed by prometamorphosis (stages 54–58), with limb growth; and ends with metamorphic climax (59–66), during which the tail is resorbed (6). The potential for tissue regeneration and motor function recovery in the *Xenopus* spinal cord is restricted to larval stages 48–54, with little recovery observed from stage 56 onwards (7–10). Thus, *Xenopus* provides an ideal model for the study of regenerative processes, including spinal cord regeneration, because of the possibility to perform intraspecies comparisons that can allow the identification of factors that allow regeneration in the tadpole, and those that inhibit regeneration in the juvenile froglet.

During the past years, there have been a few approaches to obtain a high-throughput characterization of the regenerative process of the spinal cord in *Xenopus laevis* and its closely related frog, *Xenopus tropicalis*. In 2011, the Amaya group reported the transcriptome of tail regeneration in the *X. tropicalis* larva (11), which allowed identifying reactive oxygen species as key players during tail regeneration (12). Also in 2011, the Szaro group reported the transcriptome after spinal cord transection in regeneration-permissive and regeneration-inhibiting conditions through the modulation of thyroid hormone levels, which play a key role in driving metamorphosis (13). In a more recent report we used RNA-Seq to obtain the transcriptome in response to spinal cord transection comparing a regenerative and a nonregenerative stage, 1, 2, and 6 days after injury (8). Interestingly, the repertoire of transcripts regulated in response to spinal cord transection is strikingly different when comparing regenerative and nonregenerative stages, with only 19% differentially expressed in both stages after SCI. Also, the regenerative stage differentially expresses a large number of transcripts 1 day after transection, whereas the nonregenerative stage shows a delayed equivalent response only 6 days after transection. Furthermore, transcripts related to neurogenesis, axonal regeneration, metabolism, immune response and development, among others, show a different response to SCI when comparing the regenerative and nonregenerative stage (8).

Although a transcriptomic approach delivers valuable information, mRNA and protein levels do not always correlate, as these depend on protein half-life, regulated by translation and degradation rates. In fact, in the developing embryo, less abundant proteins with short half-lives show a higher correlation with their mRNA levels than their more abundant, longer half-life counterparts (14). Thus, a proteomic profile of the response to spinal cord injury in *Xenopus laevis* can contribute different and important information regarding the mechanisms that govern spinal cord regeneration.

Quantitative proteomics of *X. laevis* eggs (15, 16) and early development embryos (14, 17, 18) has been reported. Although several studies have performed quantitative proteomics after SCI in mammalian model organisms (19–22), none have addressed spinal cord regeneration in *Xenopus*.

Here, we used iTRAQ (isobaric tags for relative and absolute quantification) chemistry to obtain a quantitative proteome of the spinal cord 1 day after injury in a regenerative and a nonregenerative stage in *X. laevis*. The iTRAQ method uses isotopic labeling for protein identification and quantification of peptide fragments from low mass reporter ions at the MS/MS level (23). An important benefit is that it allows analyzing up to eight pools of peptides in a single analysis (iTRAQ 8-plex), speeding analysis as well as minimizing the error associated with running replicates or samples for which protein abundance will be compared, in separate experiments (24).

We identified 7859 protein groups and obtained quantitative data for 6384 proteins considering all samples. Differential expression analyses identified a total of 398 proteins differentially expressed in response to SCI, with 172 in the regenerative stage and 240 in the nonregenerative stage. Only 14 proteins showed the same expression change to spinal cord injury in both stages, indicating that the protein repertoire in response to injury is even more different than the transcriptional repertoire when comparing regenerative and nonregenerative stages. Gene ontology enrichment analysis also revealed that although the regenerative stage differentially expresses proteins enriched in protein transport, synapse/vesicle and mitochondria, the nonregenerative stage enriched lipid metabolism and translation/ribosomal related proteins. STRING protein-protein network analysis also allowed identification of the most highly connected nodes among differentially expressed proteins, which represent the best candidates to regulate the response to injury and the regenerative process. In summary, this is the first quantitative proteome of the response to spinal cord injury in *Xenopus laevis*, including molecular detail on the proteins that respond to injury in a regenerative and a nonregenerative stage. These results contribute to the understanding of the mechanisms involved in spinal cord regeneration in regenerative organism and provide insights for elucidating why this process fails in mammals.

## EXPERIMENTAL PROCEDURES

### 

#### 

##### Growth of Xenopus laevis Tadpoles and Froglets

Animals were obtained by *in vitro* fertilization and cultured as described (7, 8, 25) using frogs from Nasco (Fort Atkinson, WI), until they achieved Nieuwkoop and Faber (NF) stages 49–51 for the regenerative stage (R-stage), and stage 66 for the nonregenerative stage (NR-stage). Spinal cord transection was performed as previously described (8, 25). All animal procedures were approved by and carried out in accordance with the guidelines from the Comité Ético Científico para el Cuidado de Animales y Ambiente (Scientific Ethics Committee for Animal and Environmental Care, protocol #150507001) from the Pontificia Universidad Católica de Chile.

##### Spinal Cord Isolation

**Fig. 1.**
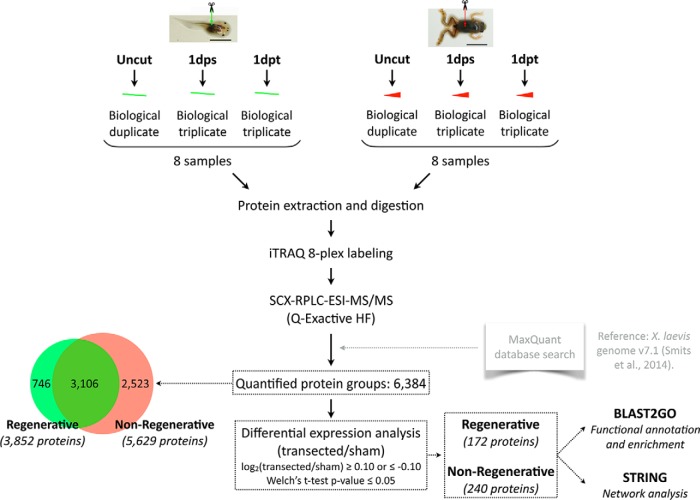
**Experimental workflow.** One iTRAQ 8-plex experiment for each stage (regenerative, left; nonregenerative, right) was performed, including duplicate uncut samples, and triplicate sham (1dps) and transected (1dpt) samples isolated 1 day after surgery. Two iTRAQ channels were used for labeling of duplicate uncut samples. Three iTRAQ channels were used for labeling of triplicate sham (1dps) samples, and the remaining three channels for triplicate transected (1dpt) samples. Approximately 0.95 million tandem mass spectra were acquired, which corresponded to over 50,000 peptide sequences, and 7859 identified protein groups. 6384 had quantifiable levels, corresponding to 3852 proteins in the regenerative stage, and 5629 proteins in the nonregenerative stage (overlap: 3106 proteins quantified in both stages). Analysis identified 172 differentially expressed proteins in the regenerative stage, and 240 proteins in the nonregenerative stage. Functional analyses were performed using BLAST2GO and STRING for differentially expressed proteins.

Spinal cords were isolated as previously described (8, 25). Briefly, regenerative and nonregenerative stage transected, sham-operated or uncut animals were sacrificed 1 day after surgery, a caudal spinal cord segment was dissected, and the tissue was immediately placed in an Eppendorf tube for flash freezing in liquid nitrogen. For the regenerative stage, each sample contained a pool of spinal cords from 5 animals, wherein biological triplicates were prepared for transected and sham-operated animals, and biological duplicates for uncut animals. For the nonregenerative stage, each sample contained a pool of 3 spinal cords, also obtaining biological triplicates for transected and sham-operated animals, and duplicates for uncut animals (Fig. 1).

##### Experimental Design and Statistical Rationale

A total of 8 samples were analyzed for the regenerative stage (R-stage), and 8 samples for the nonregenerative stage (NR-stage). For each stage, these included biological triplicates, *n* = 3 for sham, and *n* = 3 for transected samples, and biological duplicates (*n* = 2) for uncut (uninjured) samples. Uncut sample values were used as controls for normalization of sham and transected samples, and sham samples were used as a control for protein level changes caused by damage to the other tissues (skin, muscle), so that proteins with significant level changes could be attributed specifically to spinal cord injury. No technical replicates were considered necessary, given the fact that we included biological triplicates.

##### Protein Extraction, Tryptic Digestion and iTRAQ 8-plex Labeling

The spinal cord samples from regenerative and nonregenerative stage *Xenopus laevis* were prepared as follows. Each spinal cord sample was suspended in a lysis buffer containing 2% (w/v) sodium dodecyl sulfate (SDS), 100 mm Tris-HCl, complete protease inhibitor (Roche, Indianapolis, IN), pH 7.6. 100 μl of lysis buffer was used for each regenerative-stage spinal cord sample and 150 μl of lysis buffer for each nonregenerative-stage spinal cord sample. All the samples were homogenized for 1 min on ice, followed by sonication with a Branson Sonifier 250 (VWR Scientific, Batavia, IL) for 10 min on ice. After kept on ice for 1 h, the samples were centrifuged at 15,000 g for 10 min. The supernatants were collected for further experiments. A small portion of each sample was used for BCA assay to determine the protein concentration.

Based on the protein concentration, we took 20 μg of protein materials from each regenerative-stage spinal cord sample and 100 μg of protein materials from each nonregenerative-stage spinal cord sample for further preparation. All the samples were denatured at 90 °C for 15 min, followed by protein reduction with dithiothreitol at 60 °C for 1 h and alkylation with iodoacetamide at room temperature for 30 min. Each sample was then mixed with 8 m urea in 100 mm ammonium bicarbonate (pH 8) and loaded onto a filter membrane (regenerated cellulose membrane, molecular weight cut-off as 30,000 Da) integrated in the Microcon® -30 centrifugal filter unit (Merck, Darmstadt, Germany) for protein sample clean-up and tryptic digestion based on the filter aided sample preparation (FASP) protocol. Briefly, the protein materials on the membrane were washed with 8 m urea in 100 mm ammonium bicarbonate for five times to remove the SDS. Then, the proteins on the membrane were washed with 100 mm ammonium bicarbonate twice to remove urea. After centrifugation, 50 μl of 100 mm ammonium bicarbonate (pH 8) was added onto the membrane to suspend the protein materials on the membrane via gentle vortex. Then, l- (tosylamido-2-phenyl) ethyl chloromethyl ketone (TPCK)-treated trypsin (Sigma Aldrich, St. Louis, MO) was added into the protein solution for protein digestion for overnight at 37 °C with protein-to-trypsin ratio as 30/1 (w/w).

After protein digestion, each filter unit was centrifuged at 18,000 × *g* for 15 min to collect the flow-through peptides. To increase peptide recovery, another 50 μl of 100 mm ammonium bicarbonate (pH 8) was added onto the membrane, followed by centrifugation. The collected peptides from the two steps were combined. The peptide sample was then acidified with formic acid (FA) with final FA concentration as 0.5% (v/v).

All the peptide samples from regenerative and nonregenerative stages (16 in total) were desalted with C18 SPE columns and lyophilized, followed by iTRAQ 8-plex labeling based on the procedure from the manufacture (AB Sciex, Foster City) (23). Briefly, for regenerative stage samples (8 samples, 20 μg peptides/sample), each sample was re-dissolved in 10 μl of dissolution buffer and 20 μl of iTRAQ reagent was added into each sample for labeling at room temperature for 2 h. The eight samples were labeled with the different channels of iTRAQ 8-plex reagents. For the nonregenerative stage samples (eight samples, 100 μg peptides/sample), each sample was re-dissolved in 30 μl of dissolution buffer and one whole tube of iTRAQ reagent (for labeling of 100 μg of peptides) was added into each sample for labeling at room temperature for 2 h. The eight samples were labeled with the different channels of iTRAQ 8-plex reagents. For both regenerative and nonregenerative stage samples, iTRAQ channels 113 and 114 were used to label uncut samples (biological duplicate); iTRAQ channels 115, 116, and 117 were used to label sham-operated samples (biological triplicate); iTRAQ channels 118, 119, and 121 were used to label transected samples (biological triplicate). After blocking the excess iTRAQ reagent with 100 mm Tris-HCl buffer (pH 7.6), we combined the eight labeled regenerative stage samples into one sample and combined the eight labeled nonregenerative-stage samples into another sample. After lyophilization, we re-dissolved the two samples in 0.5% (v/v) FA, followed by peptide desalting with C18 SPE column and lyophilization. Finally, we dissolved the regenerative-stage sample (160 μg of iTRAQ labeled peptides) and the nonregenerative-stage sample (800 μg of iTRAQ labeled peptides) in 250 μl and 900 μl of 0.1% (v/v) FA containing 2% acetonitrile, respectively, followed by strong cation exchange (SCX) fractionation.

##### SCX Fractionation

A Waters Alliance HPLC system (Waters, Milford, MA) was used for SCX fractionation of the iTRAQ labeled peptides. The flow rate of mobile phase is 0.3 ml/min. The separation column was a Zorbax 300-SCX column (2.1 mm i.d. × 150 mm length, 5 μm particles, Agilent Technologies, Santa Clara, CA). An SCX trap column (4.6 mm i.d. × 12.5 mm length, Agilent Technologies) was connected to the separation column for the nonregenerative-stage sample fractionation.

The mobile phase gradient was generated using buffer A (8 mm KH_2_PO4, 20% ACN, pH 2.8) and buffer B (0.8 m KCl in A, pH 2.8). The samples were loaded onto the SCX column, followed by 20 min washing with 100% A to remove excess iTRAQ reagent. Then, the peptides were separated by a 60-min linear gradient from 100% A to 100% B. Finally, the column was washed with 100% B for 20 min, followed by column equilibration with 100% A. 160 μg of iTRAQ labeled peptides of regenerative-stage sample was loaded for fractionation. 700 μg of iTRAQ labeled peptides of the nonregenerative-stage sample was loaded for fractionation.

In total, 61 fractions were collected for the regenerative-stage sample and 52 fractions were collected for the nonregenerative-stage sample. After fraction combination, we obtained 23 fractions for the regenerative-stage sample and 26 fractions for the nonregenerative-stage sample. All the fractions were lyophilized and desalted. After lyophilization again, we redissolved the samples in 0.1% (v/v) FA containing 2% acetonitrile (5 μl for regenerative-stage-sample fractions and 15 μl for nonregenerative-stage-sample fractions) for reversed-phase liquid chromatography (RPLC)-electrospray ionization (ESI)-tandem mass spectrometry (MS/MS) analysis.

##### RPLC-ESI-MS/MS

A nanoACQUITY UltraPerformance LC® (UPLC®) system (Waters) was used for peptide separation. Buffer A (0.1% FA in water) and buffer B (0.1% FA in acetonitrile) were used as mobile phases for gradient separation. Peptides were automatically loaded onto a commercial C18 reversed phase column (Waters, 100 μm × 100 mm, 1.7 μm diameter particle, BEH130C18, column temperature 40 °C) with 2% buffer B for 14 min at a flow rate of 0.7 μl/min, followed by 3-step gradient separation, 1 min from 2% to 8% B and flow rate from 0.7 μl/min to 0.6 μl/min, 84 min to 28% B at a flow rate of 0.6 μl/min, 1 min to 80% B and flow rate from 0.6 μl/min to 0.7 μl/min, and maintained at 80% B for 5 min with a flow rate of 0.7 μl/min. The column was equilibrated for 14 min with 2% B at a flow rate of 0.7 μl/min before analysis of the next sample. The eluted peptides from the C18 column were pumped through a capillary tip for electrospray, and analyzed by a Q-Exactive HF mass spectrometer (Thermo Fisher Scientific, Waltham, MA). For each regenerative-stage sample, 4 μl of peptides were loaded onto the column for analysis. For each nonregenerative-stage sample, 2 μl of peptides were loaded onto the column for analysis.

The electrospray voltage was 2 kV, and the ion transfer tube temperature was 300 °C. The S-Lens RF level was 60.00. The data acquisition was programmed in data dependent acquisition (DDA) mode. A top 12 method was used for nonregenerative stage samples and a top 5 method for regenerative stage samples. Full MS scans were acquired in Orbitrap mass analyzer over *m*/*z* 350–1800 range with resolution of 60,000 (*m*/*z* 200) and the number of microscans set to 1. The target value was 3.00E+06. For MS/MS scans, the twelve (for nonregenerative stage) or five (for regenerative stage) most intense peaks with charge state ≥ 2 were sequentially isolated and further fragmentation in the higher-energy-collisional-dissociation (HCD) cell following one full MS scan. The isolation window was set as 1.2 *m*/*z*. The normalized collision energy was 30%, and tandem mass spectra were acquired in the Orbitrap mass analyzer with resolution 60,000 (*m*/*z* 200). The fixed first mass was m/z 100.0. The target value was 1.00E+05 and maximum injection time was 110 ms (for nonregenerative stage) or 200 ms (for regenerative stage). The number of microscans was 1 and the ion selection threshold was 1.0E+05 counts. Peptide match was off and exclude isotopes was turned on. Dynamic exclusion was set as 60 s. Of note, the protein material used in the experiment for the regenerative stage was much lower than that for the nonregenerative stage because of the limited mass of proteins in each spinal cord at the regenerative stage, which is why increased the MS2 injection time to 200 ms for the regenerative stage, and we used a Top 5 method to control the cycle time, in order to improve the protein identification and quantification.

##### Protein Identification and Quantification

All the raw files were analyzed with MaxQuant software version 1.5.2.8 (26). The protein reference database from *X. laevis* genome v7.1 was used for database searching (15). Variable modifications were acetyl (Protein N-term), deamidation (NQ), and oxidation (M). Carbamidomethyl (C) was set as a fixed modification. Trypsin with specific digestion was applied. For the iTRAQ quantification, the “Reporter ion MS2” function was chosen. iTRAQ 8plex-Lys 113–121 and iTRAQ 8plex-Nterm 113–121 were used. Other iTRAQ related parameters were as follows: reporter mass tolerance as 0.01 Da, filter by precursor intensity fraction (PIF) with minimal reporter PIF as 0.75. Other parameters were default settings. The *X. laevis* genome v7.1 database contains 54,130 protein sequences. The maximum number of allowed missed cleavages for database search was two. The mass tolerance for parent ions was 20 ppm for the first search and 4.5 ppm for the main search. The mass tolerance for fragment ions was 20 ppm. The common contaminants were included in the database for search and the identifications corresponding to the contaminants were removed from the final identification lists. The database searching results were filtered with false discovery rates (FDRs) less than 1% on both peptide and protein levels. During the database search, all the protein identification and quantification information from raw files of the regenerative stage were merged; the information from raw files of nonregenerative stage were merged. Finally, we got one file containing the protein identification and quantification information for both stages. In the file, MaxQuant reported the reporter ion intensity of each iTRAQ channel for each protein, representing the abundance of proteins in different conditions. We further processed the data with Perseus software to normalize the data and to perform statistical analysis. Data for all reporter ion intensities, before and after normalization, have been included in supplemental Data S3. See supplemental Data S4 for a protein group and peptide ID list.

The export file of MaxQuant software was loaded into the Perseus software. First, we filtered the data to remove the proteins identified from reverse database and contaminant proteins. Second, we averaged the reporter ion intensity of the biological duplicate of uncut samples for both regenerative and nonregenerative stages. Third, we normalized the reporter ion intensity of transected and sham-operated samples to the averaged uncut samples for both stages to get the protein expression ratios compared with uncut samples. Then, we performed bias correction for protein quantitation results in Perseus with the “divide” function. Briefly, the median protein ratio in each biological condition (biological triplicates of transected and sham-operated) was corrected to unity, and then this factor was applied to all quantitation results in each corresponding biological condition. Finally, we performed differential expression analyses in Perseus to compare the protein expression in transected and sham-operated samples. We used the “Two-sample tests” function, which allowed performing a modified *t* test to determine whether the means of biological triplicates of transected and sham-operated samples were significantly different (*p* value < 0.05). The “first group (right)” was assigned to transected samples, and the second group was assigned to sham-operated samples using the “specify individual groups” option. Welch's *t* test was selected in “Test,” using S0 = 0; Side = Both, and “*p* value” was selected in the “Use for truncation” option, with a 0.05 threshold. The remaining parameters were left as default. Results with significant changes (*p* value < 0.05) were then additionally filtered by fold-change: log_2_ (transected/sham) ≥ 0.10 or ≤ −0.10. It is important to note that we used the nominal *p* value to determine significance, and that when we performed multiple hypothesis testing using the Benjamini-Hochberg correction (threshold = 0.05), we did not obtain any significant differential expression changes.

##### Gene Ontology Enrichment

Blast2GO (27) was used to perform gene ontology (GO) enrichment for proteins that met differential expression criteria. Peptide sequences for each protein were used to perform BLAST was performed using the following criteria: “Blast DB: nr,” “Taxonomy filter: vertebrates (taxa: 7742,Vertebrata),” “E-value cut-off: 1.0E-3′. As a background for the Fisher”s exact test, we used the same UniProtKB database generated previously (8). GO terms with a false discovery rate (FDR) < 0.05 were obtained, and those belonging to level 5 were then selected and included in supplemental Data S2. A Venn diagram with all level 5 terms was then constructed for each category (Biological Process, Molecular Function, Cellular Component) using BioVenn (28), and only terms exclusively found in either stage were selected. These were ranked according to their FDR value (ascending), and top 10 terms for Biological Process and Cellular Component were selected for the main figure (Fig. 4). A full list of exclusive level 5 terms (including Molecular Function) for each stage is included in supplemental Data S2.

##### Heatmap Generation and Clustering

Proteins belonging to the GO terms listed in Fig. 5 were selected for clustering and heatmap construction using their log_2_ (transected/sham) fold-change. Relevant GO terms were manually grouped into five categories: “Protein transport,” “Synapse/Vesicle,” “Mitochondria,” “Lipid metabolism” and “Translation/Ribosomal.” Hierarchical clustering for each group was performed with Cluster 3.0 (29), using the “Correlation (centered)” similarity metric, and the “Average linkage” clustering method. Output.cdt files were used to generate heatmaps using Java TreeView (30), as previously reported (8).

##### STRING Analysis

Differentially expressed proteins from each stage were analyzed using STRING (31). This database does not include data for *Xenopus laevis*, although it does contain data for *Xenopus tropicalis*. However, the human database was selected for analysis as it contained substantially more information than that for *X. tropicalis*. Gene symbols for each protein were used to find human orthologues and generate STRING networks using default settings. XML files with interaction data were then exported and processed using Cytoscape.

##### Comparison of Transcriptomic and Proteomic Gene Ontology Enrichment Analyses

Lists for all gene ontology (GO) terms enriched in our previous transcriptomic data (8) for “biological process” and “cellular component,” 1 day after spinal cord injury, for regenerative and nonregenerative stages were generated (four lists in total). Equivalent lists were generated from the GO enrichment analysis in the present proteomic analysis (four additional lists). This allowed the generation of Venn diagrams of the GO terms enriched at both the transcriptome and proteome levels (four Venn diagrams, and therefore, four intersection lists of GO terms). This list was then filtered to select only level 5 GO terms (according to the proteome GO enrichment analysis), with their corresponding false discovery rate (FDR) values. A final Venn diagram was obtained to generate a list of GO terms exclusively enriched in each stage. In summary, this analysis allowed us to generate lists of GO terms enriched: (1) At both the transcriptome and proteome level; (2) Slimmed to select only 'level 5' terms; and (3) Enriched exclusively in either the regenerative or in the nonregenerative stage.

## RESULTS

### 

#### 

##### Quantitative Proteomic Analysis of Xenopus laevis Spinal Cord After Injury

In a previous report, we found that the transcriptome of the spinal cord in regenerative (stage 50) *Xenopus* tadpoles responds with differential expression of many transcripts at only 1 day after transection. Instead, nonregenerative froglets (stage 66), differentially express a much lower number of transcripts 1 day after transection, but this number increases substantially 6 days after transection (8). This difference in timing made the 1 day after transection time point of interest to study at the protein level. We therefore performed transection and sham surgeries on regenerative and nonregenerative stage animals, and isolated the caudal segment of the spinal cord 1 day after surgery, obtaining an equivalent sample to that used for our previous transcriptome study. Samples were prepared in biological triplicates, with each containing a pool of 5 regenerative stage spinal cords, or 3 nonregenerative stage spinal cords. Samples for either stage were obtained from the same clutch to decrease biological variability. Pooling for each sample ensured obtaining a representative sample, and triplicates allowed accounting for biological reproducibility. In addition to spinal cords from operated animals, we also included a biological duplicate for uninjured animals (uncut samples) (Fig. 1).

Regenerative and nonregenerative stage samples were labeled separately using 8-plex iTRAQ (isobaric tags for relative and absolute quantification) reagents, and peptides were pooled and separated by strong cation exchange liquid chromatography, allowing for separate runs for each stage, followed by RPLC-ESI-MS/MS analysis with a Q-Exactive HF mass spectrometer. Data was analyzed using MaxQuant and a reference database based on the *X. laevis* genome v7.1 (15).

Our analyses allowed the identification of 7859 protein groups and the quantification of 81% of these, constituting a total of 6384 proteins (for a full list of quantified proteins refer to supplemental Data S1). 3852 proteins were quantified in the regenerative stage and 5629 in the nonregenerative stage, with an overlap of 3106 proteins (Fig. 1). Details supporting the reproducibility of our data have been included in supplemental Data S5. A high correlation among uncut samples was found for both stages (*r* = 0.99; slope 0.98–1.0) (supplemental Fig. S1-S2), and median values for the relative standard deviation (RSD) ranged between 8 and 17% (supplemental Fig. S3, S5, S6) for sham and transected samples. The only exception was the regenerative stage transected sample, which showed a higher RSD of 35% (supplemental Fig. S4). This higher variance may be explained by the larger percentage of differential expression at the transcriptome level during the first phases of the response to injury (8). Given the rapid nature of this response, it is expected that there will be a larger inter-individual variation, as opposed to the low variance observed in uncut samples, which have not received any kind of noxious stimuli.

**Fig. 2.**
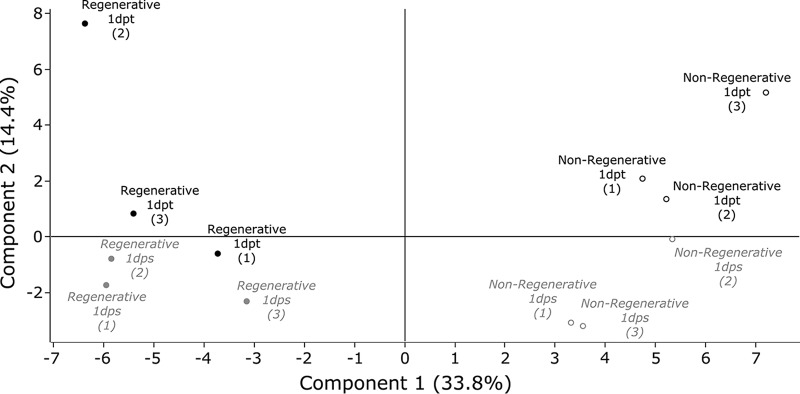
**Principal Component Analysis.** Reporter ion intensities for transected and sham operated samples were normalized against uncut samples, and principal component analysis performed on normalized values. Regenerative (●) and nonregenerative stage (○) samples were separated along component 1, and 1 day after transection (1dpt, black) and 1 day after sham-operation (*1dps, gray*) samples separated along component 2. Percentages (%) indicate the percentage of variance that each principal component represents.

Reporter ion intensities for transected and sham samples were then normalized against the mean value of the uncut duplicate samples (see Methods), allowing a normalization against the baseline levels for each protein in the uninjured animal. Principal component analysis showed that regenerative and nonregenerative stage samples distributed along component 1, supporting the notion that the proteomes expressed by both stages were vastly different when compared with one another (Fig. 2). Importantly, the second component separated transected from sham samples, indicating that specific changes at the proteome level can be detected 1 day after spinal cord injury.

Overall, we have obtained high quality and reproducible quantitative data for the proteins expressed in the spinal cord in an injury context, including biological triplicates for sham and transected samples, which we additionally normalized against the uncut samples for increased robustness.

##### Differential Expression of Proteins in Response to Spinal Cord Injury

To identify the proteins that changed their levels in response to spinal cord transection, we performed differential expression analysis of normalized transected and sham reporter ion intensities to obtain a transected/sham fold-change (see Methods). We performed this analysis for all proteins quantified in either stage (regenerative or nonregenerative). For this purpose, we used a modified Student's *t* test (Welch's, nominal *p* value < 0.05), and included an additional fold-change filter (log_2_fold-change ≥ 0.10 or log_2_fold-change ≤ −0.10) for selecting differentially expressed proteins. It is worth noting that we chose to use a relatively low threshold for the fold-change, given that the goal of this study was to identify biological processes that may play a role in spinal cord regeneration, wherein small fold-changes of a group of proteins involved in the same process could have a significant effect. Therefore, although we will address differential protein expression, our focus will be on gene ontology enrichment analyses in the following section.

**Fig. 3.**
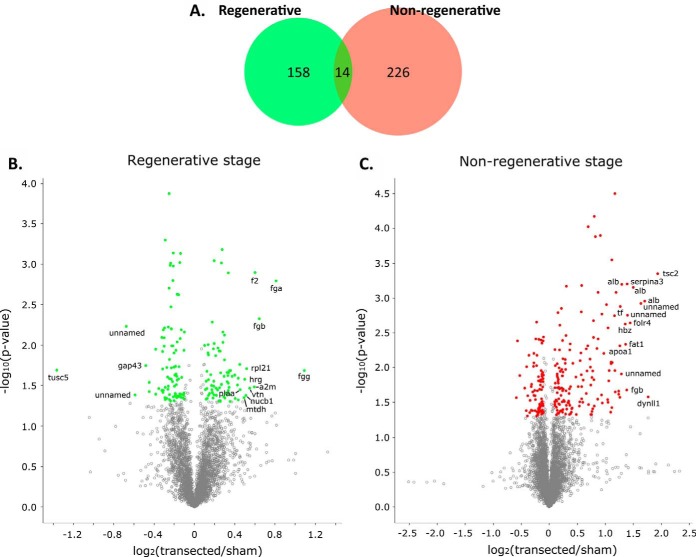
**Proteins showing differential expression when comparing transected and sham-operated animals.** Differentially expressed proteins were determined using a Welch's *t* test (*p* value < 0.05) and an additional log_2_ (transected/sham) ≥ 0.10 or ≤ −0.10 fold-change filter. *A*, Venn diagram showing the number of proteins meeting differential expression criteria for each stage, distributed into those which did so in the regenerative, nonregenerative stage, or both. *B*, *C*, Volcano plots showing log_2_ (transected/sham) fold-change (x-axis) and -log_10_ (*p* value) (x-axis) for all quantified proteins in the regenerative (*B*) or the nonregenerative stage (*C*). Colored dots indicate proteins meeting differential expression criteria, and labels show gene symbols for top 15 proteins with highest fold-change. *Regenerative- green; nonregenerative- red*.

We identified 172 differentially expressed proteins in the regenerative stage, and 240 proteins in the nonregenerative stage (see supplemental Data S1 for detailed list of differentially expressed proteins). A comparison among differentially expressed proteins in either stage indicated that only 14 proteins (4%) were differentially expressed in both stages (Fig. 3*A*), demonstrating that regenerative and nonregenerative stages regulate different repertoires of proteins in response to spinal cord transection. These 14 proteins were all upregulated in both stages (data not shown). Regarding differentially expressed proteins, although we cannot perform a direct comparison to determine whether they were expressed in a stage-specific manner (because of different initial protein quantities and therefore, differing coverage, as well as experiments for each stage having been performed in separate iTRAQ 8-plex runs), we found that in the regenerative stage, 140/172 differentially expressed proteins were also quantified in the nonregenerative stage. In the nonregenerative stage, 105/240 differentially expressed proteins were also quantified in the regenerative stage, which may be because of the larger coverage obtained for this stage. It is therefore likely that instead of stage-specific protein expression, what we observe is instead stage-specific protein regulation.

Furthermore, the regenerative stage up and downregulates a similar number of proteins (81 and 91, respectively), whereas the nonregenerative stage upregulates twice as much proteins as the ones it downregulates (155 and 85, respectively; Fig. 3*B*). Of note, the magnitude of the protein fold-change after transection shown by the nonregenerative stage was higher than that observed in the regenerative stage (Fig. 3*B–C*, note X-axis for each graph). Proteins with the largest differential expression changes (top 15) included Tumor suppressor candidate 5 (Tusc5) and growth associated protein 43 (Gap43) in the regenerative stage, whereas the nonregenerative stage included tuberous sclerosis 2 (Tsc2) from the TOR signaling pathway and albumin (Fig. 3*B–C*; supplemental Data S1).

##### Gene Ontology Enrichment of Differentially Expressed Proteins

**Fig. 4.**
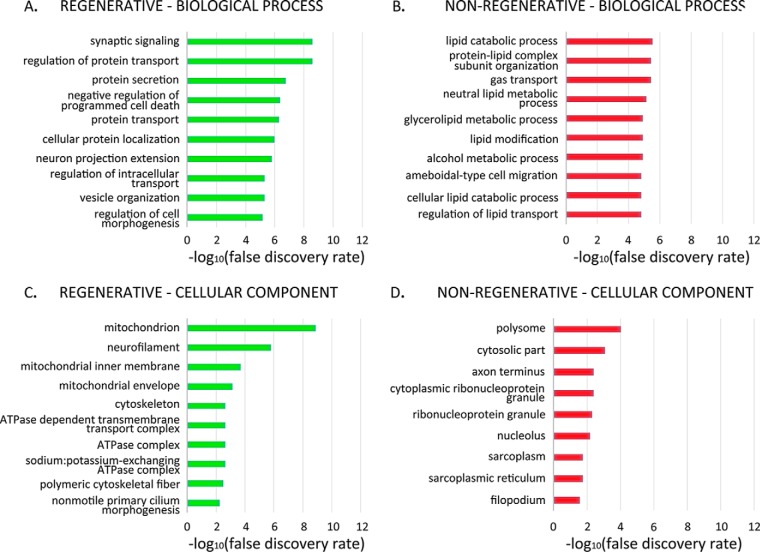
**Gene ontology enrichment for differentially expressed proteins in the regenerative and nonregenerative stage.** Gene ontology (GO) enrichment analysis was performed for proteins meeting differential expression criteria (see Fig. 3 legend) using Fisher's Exact Test, reporting terms with a false discovery rate (FDR) < 0.05. In these graphs, terms were additionally filtered so that only terms exclusively found in one stage but not the other are shown. Top 10 categories with the lowest FDR are shown when more than 10 exclusive GO terms were found.

We next performed gene ontology (GO) annotation and enrichment analysis for the sets of differentially expressed proteins, performing separate analyses for each stage. To maximize the information available for our protein set, we used BLAST2GO to match the GO annotation of our dataset with that available for all vertebrate species (see Methods). Once protein annotation was complete, we performed GO enrichment analysis using a Fisher's Exact Test. Level 5 GO terms were selected, and only those found exclusively enriched in either stage were included in the graphs. When more than ten exclusive terms were found, only those with the lowest false discovery rate were included in the bar graphs (Fig. 4). A full list of all enriched terms from biological process, cellular component and molecular function is included in supplemental Data S2, as well as a full list of level 5 GO terms. A selection of relevant GO terms in each category is described below.

**Fig. 5.**
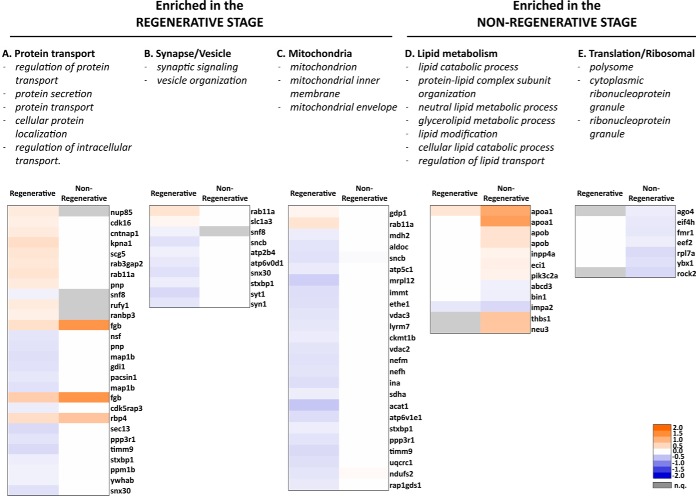
**Heatmaps showing differential expression changes for proteins belonging to enriched gene ontology (GO) categories.** Gene ontology terms were classified into 5 groups (*A–E*). Specific GO terms included in each group are listed below. n.q. protein was not quantified (gray boxes). Color scale represents log_2_ (transected/sham) fold-change values with significant differential expression (p value < 0.05). Fold-changes with nonsignificant p value (≥ 0.05) are shown in white.

In the regenerative stage in the biological process category, 5/10 terms enriched were related to protein transport, including “regulation of protein transport,” “protein secretion,” “protein transport,” “cellular protein localization,” and “regulation of intracellular transport” (Fig. 4*A*). These proteins were similarly distributed between up and downregulated after transection in the regenerative stage, including among others intracellular trafficking proteins Rab11a (RAB11A, member of the RAS oncogene family), Rab3gap2 (RAB3 GTPase activating noncatalytic protein subunit 2) and Sec13 (SEC13 homolog, nuclear pore and COPII coat complex component) (Fig. 5*A*). Interestingly, 2/10 terms were related to synapse/vesicle: “synaptic signaling” and “vesicle organization” (Fig. 4*A*). These proteins were mostly downregulated after spinal cord transection in the regenerative stage, and remained unchanged in the nonregenerative stage, and included Syn1 (Synapsin I), Syt1 (Synaptotagmin 1), and Stxbp1 (Syntaxin binding protein 1) (Fig. 5*B*).

In addition, the following terms related to mitochondria were found enriched in the regenerative stage: “mitochondrion,” “mitochondrial inner membrane,” “mitochondrial envelope” (Fig. 4*C*). In this category, except for 2 proteins, all were significantly downregulated (*p* value < 0.05) after spinal cord transection in the regenerative stage, while showing no significant change in the nonregenerative stage, including mitochondrial outer membrane channels Vdac2/3 (voltage-dependent anion channel 2/3), and the ATP synthase subunit Atp5c1 (ATP synthase, H+ transporting, mitochondrial F1 complex, gamma polypeptide 1) (Fig. 5*C*).

The nonregenerative stage enriched 7/10 terms related to lipid metabolism and transport, including “lipid catabolic process,” “protein-lipid complex subunit organization,” “neutral lipid metabolic process,” “glycerolipid metabolic process,” “lipid modification,” “cellular lipid catabolic process” and “regulation of lipid transport” (Fig. 4*B*). These proteins were mostly upregulated after transection (Fig. 5*D*).

Also in the nonregenerative stage, 3/10 terms enriched in this category were related to translation/ribosomal, and included “polysome,” “cytoplasmic ribonucleoprotein granule,” “ribonucleoprotein granule” (Fig. 4*D*). These proteins were downregulated after transection in the nonregenerative stage, and included Ybx1 (Y-box binding protein 1), which regulates alternative splicing, and Ago4 (argonaute 4, RISC catalytic component) (Fig. 5*E*).

##### Protein-Protein Interaction Networks and Hub Nodes

**Fig. 6.**
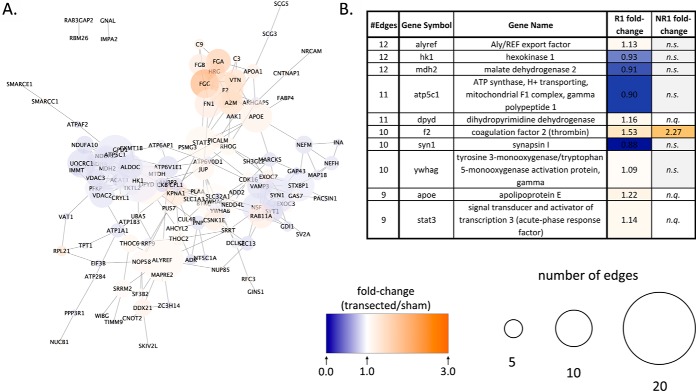
**STRING network analysis for differentially expressed proteins in the R-stage.** STRING network analysis for proteins meeting differential expression criteria (see Fig. 3 legend) in the regenerative stage. *A*, STRING network. Fill color represents fold-change, and node size represents the number of edges (or undirected connections) each protein has. *B*, List of most highly connected proteins and their *See both scales in bottom-right corner*.

**Fig. 7.**
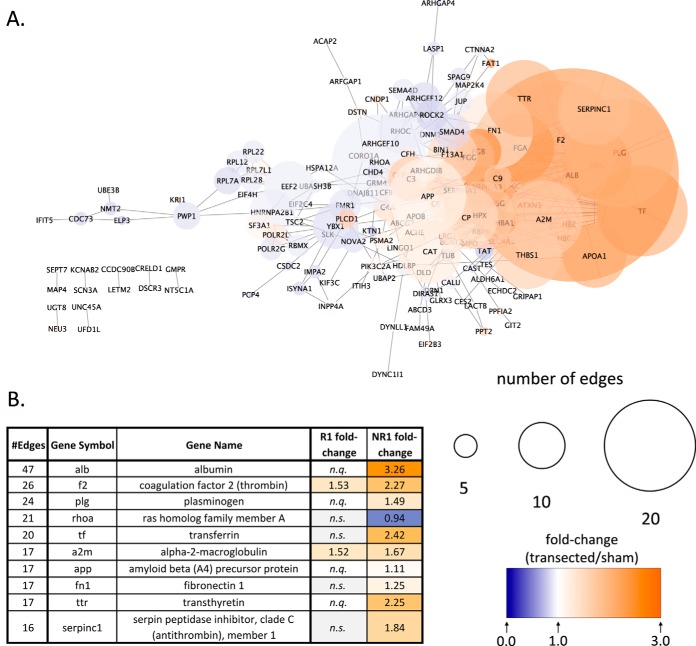
**STRING network analysis for differentially expressed proteins in the NR-stage.** See Fig. 6 for legend.

The STRING database (31) allows construction of protein-protein interaction networks, ranging from direct protein-protein interactions to indirect interactions (such as coexpression and text mining). We constructed STRING networks for the regenerative and nonregenerative stage, using only differentially expressed proteins (Figs. 6 and 7).

The network generated for the regenerative stage connected most differentially expressed proteins into a single network. However, within the network, proteins were distributed into three main clusters. The first included several proteins related to blood coagulation (Fig. 6*A*, top-right); the second, several energy metabolism related proteins, including hexokinase 1 (HK1), which is the first glycolysis enzyme, and mitochondrial voltage-gated anion channels (Vdac2/3) (Fig. 6*A*, middle-left); the third cluster included proteins involved in intracellular trafficking, such as neurofilaments (NEFM, NEFH), Rab11a, Exoc3/7, and synaptic or axonal growth cone proteins (Syn1 and Gap43) (Fig. 6*A*, middle-right). It is worth noting that the second and third clusters were mainly downregulated after transection, whereas the blood coagulation cluster was upregulated.

The most highly connected R-stage node was the Aly/REF export factor, with a 1.13 upregulation fold-change (*p* value < 0.05) after transection and no significant change detected in the NR-stage. The following three most connected proteins are all involved in energy metabolism (Fig. 6*B*): the previously mentioned glycolytic enzyme Hexokinase 1, the tricarboxylic acid cycle (TCA) cycle Malate dehydrogenase 2 (Mdh2), and the also previously mentioned ATP synthase subunit Atp5c1, all of which were downregulated in response to transection, but remained unchanged in the NR-stage.

The network for the nonregenerative stage showed a different distribution to the regenerative stage. Although most proteins were connected into a single network, the most prominent cluster within it included blood coagulation proteins (Fig. 7*A*, right, *e.g.,* fibrinogen (Fga, Fgb, Fgg), complement factor 9 (C9), transferrin (TF), etc.). There was also a smaller cluster of ribosomal proteins (Fig. 7*A*, middle-left, including Rpl12/22/7A/7D/28).

**Fig. 8.**
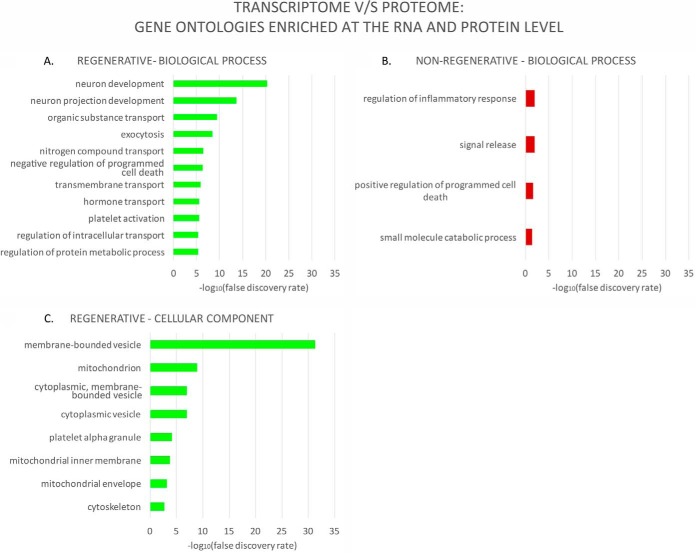
**Gene ontology (GO) categories enriched at both the transcriptome and proteome levels.** A list of GO terms enriched among differentially expressed genes, in both previously published RNA-Seq data (8) and the present iTRAQ data was generated, after which level 5 GO terms were selected and their enrichment false discovery rate (FDR) value obtained from iTRAQ data. As in Fig. 4, terms were also filtered to include only level 5 terms exclusively found in either stage. When more than 10 exclusive GO terms were found, only the top 10 with the lowest FDR value were included in the graph (see Table I and supplemental data 6 for additional information). Note: cellular component for the nonregenerative stage is not shown because there were no level 5 terms exclusively found in this stage in this analysis.

In the NR-stage, the most interconnected node was the protein Albumin (Alb), which had a 3.26-fold upregulation in response to transection, but was not quantified in the R-stage (Fig. 8*B*). Other highly connected nodes in the table were linked to blood coagulation, including coagulation factor 2 (thrombin) (F2) and plasminogen (Plg). Worth noting was amyloid beta (A4) precursor protein (App), which showed a 1.11-fold upregulation after transection and had 17 protein-protein interactions.

##### A Comparative Analysis of Gene Ontologies Regulated at the Transcriptome and Proteome Level

As reported in previous transcriptome and proteome comparative studies (for example, the Gygi and Kirschner groups (14) performed a direct comparison during *Xenopus* embryonic development), a direct gene-by-gene comparison among our previous transcriptomic analysis of the response to spinal cord injury with our current proteomic data yielded a very low number of genes displaying the same differential expression (data not shown). Although our two experiments were not designed to be compared directly, we did find 15–28% gene ontology categories enriched at both the RNA and protein levels (Table I). As in the GO enrichment analysis shown in Fig. 4, we selected level 5 GO terms from the RNA and iTRAQ intersection list, and selected categories exclusively enriched in either stage (for a full list see supplemental Data S6). In the regenerative stage, several of the top enriched GO terms found in the proteomics analysis were also enriched at the transcriptome level. These included, for biological process, the categories of “negative regulation of programmed cell death” and “regulation of intracellular transport” (Fig. 4*A* and Fig. 8*A*), which was included in the “protein transport” group in Fig. 5*A*. For cellular component, we found all the mitochondria-related terms, also exclusively enriched in the regenerative stage (Fig. 5*C* and Fig. 8*C*). In the nonregenerative stage, we found the term “regulation of inflammatory response,” which was only enriched in this stage at both the transcriptome and proteome levels (Fig. 8*B*). These and the full list of the GO term intersection between the two experiments (supplemental Data S6), strengthens the robustness of our results, wherein our findings of the biological processes regulated after spinal cord injury agreed at the transcriptome and proteome levels.

**Table I:** A comparison of the gene ontology (GO) categories enriched after spinal cord injury at the transcriptome (RNA-Seq) (8) and proteome (iTRAQ) levels. The total number of GO terms enriched among differentially expressed genes 1 day after injury, in the “biological process” and “cellular component” categories were included. A Venn diagram (not shown) was generated to determine which terms were enriched at both the RNA and protein levels, and which were only found in either analysis. Absolute number of terms (# terms) and percentages are included in the table (see Fig. 8 and supplementary Data S6 for additional information)

GO term distribution	Regenerative				Nonregenerative			
	Biological process		Cellular component		Biological process		Cellular component	
	# terms	Percentage	# terms	Percentage	# terms	Percentage	# terms	Percentage
Total GO terms	1396	100.0%	211	100.0%	857	100.0%	107	100.0%
iTRAQ only	491	35.2%	81	38.4%	585	68.3%	78	72.9%
RNA-Seq only	567	40.6%	69	32.7%	139	16.2%	12	11.2%
**Enriched in both**	**310**	**22.2%**	**61**	**28.9%**	**133**	**15.5%**	**17**	**15.9%**

## DISCUSSION

We report the first quantitative proteome of *Xenopus laevis* spinal cord tissue, comparing the response to transection injury in a regenerative and a nonregenerative stage. We successfully quantified over 6300 proteins in total when considering both stages and all conditions (transection, sham and uninjured), which was roughly 2000 more proteins than our previous study in *Xenopus* embryos (17). These proteins correspond to one-third of the genes detected using RNA-Seq transcriptomics (8), and the RNA-to-protein ratio is similar to that obtained in early stages of development in *Xenopus* embryos (14). Through this work, we: (1) Determined that the spinal cord proteomes of the regenerative and nonregenerative stage show substantial differences, in basal conditions and in response to injury; (2) Determined that differentially expressed proteins after spinal cord injury (SCI) also differed greatly between both stages, supporting that the identified proteins can be of great value in understanding spinal cord regeneration; (3) Identified specific biological processes that showed differential regulation when comparing a regeneration-competent and a regeneration-incompetent spinal cord, such as upregulation of protein transport and downregulation of synaptic and mitochondrial proteins in the regenerative stage, whereas the nonregenerative stage upregulated proteins involved in lipid metabolism but showed downregulation of translation/ribosomal proteins; (4) Determined that these proteins also have documented interactions among them, further supporting their participation as a putative network during spinal cord regeneration.

The ability to promote axon regeneration constitutes one possible therapeutical approach to improve spinal cord regeneration. Recently Tedeschi and colleagues (32) demonstrated that synaptic elements can have an inhibitory effect on axon regeneration. In their study, they identified the alpha2delta2 accessory subunit of voltage-gated calcium channels (VGCCs) as inhibitory of axon growth. This alpha2delta2 subunit regulates synaptic VGCC density and thus, vesicular release (33). They show that they are able to enhance axon regeneration after spinal cord injury using a pharmacological inhibitor of this channel (32). Here, we present evidence showing that several proteins involved in synaptic signaling and vesicle organization were downregulated in the regenerative stage, but not in the nonregenerative stage (Fig. 5*B*), supporting the notion that downregulation of synaptic proteins could facilitate axon regeneration.

Also important for axon regeneration was the detection of proteins involved in intracellular membrane trafficking. We detected differential expression of these proteins in the regenerative stage, which included Rab3gap2 (a rab3 GTPase activating complex) and Rab11a, involved in endosomal trafficking (Fig. 5*A*). After neurons are reprogrammed from a “mature” state into a regenerative-permissive or “immature” state, there is a requirement for membranes and transport of growth factor receptors to the axonal growth cone in order to allow axonal extension. These processes are mediated by endosomal trafficking (reviewed by Hausott & Klimaschewski (34)). In contrast, the nonregenerative stage did not show a major differential expression of protein transport proteins. In fact, ribosomal proteins involved in translation and ribonucleoproteins were downregulated (Fig. 5*E*), which suggests that although the regenerative stage promoted protein trafficking, the nonregenerative stage could be in a halted state.

Another remarkable group enriched exclusively in the regenerative stage included mitochondria-related gene ontology terms (Fig. 5*C*). Notably, most of these proteins were downregulated after SCI in the regenerative stage, but remained unchanged in the nonregenerative stage. Although further studies are required to determine whether this downregulation is accompanied by a decrease in total mitochondrial number, changes in morphology, or simply decrease in protein content, one possibility is that it is a neuroprotective mechanism. A decrease in oxidative activity in the mitochondria also decreases the formation of reactive oxygen species (ROS), which may be harmful to the cell and induce apoptosis (35). It has been reported that after traumatic brain injury in mice, upregulation of a mitochondrial uncoupling protein has a neuroprotective effect (36). Uncoupling proteins dissipate mitochondrial membrane potential and thus have an overall effect on diminishing ROS levels (37). The main source of ROS inside the cell is the electron transport chain. In particular, we found that the regenerative stage responded to SCI with a significant downregulation of mitochondrial channels Vdac2/3, through which most metabolites cross in and out of the mitochondrion including glycolytic pyruvate (38). Previous reports show that *knock-down* of these channels in HepG2 cells leads to a decreased mitochondrial membrane potential because of the limited entry of metabolites into the mitochondria for oxidative phosphorylation (39). Therefore, Vdac downregulation could have a neuroprotective effect in the regenerative stage spinal cord.

Another mechanism involved in spinal cord regeneration is neurogenesis, which involves the activation of neural stem and progenitor cells (NSPC) in response to injury. We previously found that spinal cord transection induces massive activation of Sox2^+^ NSPC in regenerative tadpoles (7, 9). Recent work has shown that embryonic stem cell metabolic flux is affected by nutrient availability, in particular, culturing cells in media with abundant lipid content causes glucose and glutamine flux to be directed toward oxidative mitochondrial metabolism, while culturing in low-lipid levels prioritizes anaerobic ATP synthesis and upregulation of lipid synthesis (40). Here, we found that the nonregenerative stage responded to injury by upregulating a group of lipid metabolism proteins (Fig. 5*E*), which could suggest that lipid availability is abundant in the nonregenerative stage. This could result in augmented mitochondrial metabolism and production of ROS, which is opposite to what happens in the regenerative stage, with the possible downregulation of mitochondrial oxidative activity.

The results found in our present proteomic study were in agreement with our previous transcriptome data (8) at three different levels. First, they both showed largely different repertoires of differentially expressed genes after SCI: only 18.9% of transcripts were regulated in both stages considering all measured time-points (1, 2, and 6 days after injury), and at the protein level, less than 4% were regulated in both stages, strongly supporting that the response to SCI is substantially different when comparing the regenerative and nonregenerative stage.

Second, a comparison of gene ontology (GO) enrichment analysis results for differentially expressed genes at the transcript and protein level yielded a substantial percentage (15–28%) of categories in common, despite our experiments not having been designed for direct comparison. These included some of the top enriched GO terms that were found specifically in the regenerative stage, like “regulation of intracellular transport,” “negative regulation of programmed cell death,” and three GO terms related to mitochondria. In the nonregenerative stage we also found “regulation of inflammatory response,” which in our transcriptomic data was one of the main biological processes found upregulated in nonregenerative animals, but not in regenerative animals.

Third, if we interpret the biological significance integrating transcript and protein functional enrichment, the regenerative stage displays an overall neuroprotective environment, as supported by: a transient upregulation of response to stress transcripts, coupled with downregulation of cell death and oxidation-reduction transcripts, coupled to a massive downregulation of mitochondrial proteins, which, as discussed previously, may also play a role in decreasing oxidative stress, contributing to a neuroprotective effect too. In contrast, the nonregenerative stage upregulates transcripts involved in the immune response and inflammation, and displays a sustained upregulation of response to stress transcripts. At the protein level, the upregulation of lipid metabolism genes might lead to increased oxidative stress, which could have a potential involvement in the increase in response to stress and inflammation transcripts these are possibly detrimental responses to spinal cord injury, leading to an increase of cell death instead of neuroprotection as seen in the regenerative stage. This comparison and integration of transcriptome and proteome results at these three levels further supports the robustness of our data and the value of our contribution as a database for spinal cord regeneration in *Xenopus laevis.*

The work presented here provides further detail into the molecular mechanisms of the early response to SCI. The data is not only in agreement with our previous transcriptome experiment, but also provides additional information, such as the identification of an early downregulation of mitochondrial and synaptic vesicle proteins in the regenerative stage, which we had not detected at the transcript level. It is worth noting that the number of genes detected using proteomics was substantially lower than that obtained using RNA-Seq, making it less likely to detect lower abundance proteins such as transcription factors. The repertoire of proteins and biological processes that we have identified to have different expression changes to SCI when comparing a regenerative and a nonregenerative stage in *Xenopus* represent excellent candidates for future functional studies for identifying novel mechanisms that promote or inhibit spinal cord regeneration.

## DATA AVAILABILITY

The mass spectrometry proteomics data have been deposited to the ProteomeXchange Consortium via the PRIDE(41) partner repository with the dataset identifier PXD006993, available at: http://www.proteomexchange.org/.

## Supplementary Material

Supplemental Data

## Footnotes

^1^ The abbreviations used are:

SCI: Spinal cord injury
nr: Nonredundant protein sequence database
BLAST: Basic Local Alignment Search Tool
CNS: Central nervous system
dps: days post sham operation
dpt: days post transection
GO: gene ontology
iTRAQ: isobaric tags for relative and absolute quantification
MS222: ethyl 3-aminobenzoate methanesulfonate
NF: Nieuwkoop and Faber staging for *Xenopus laevis* development
NR: Nonregenerative
NSPC: neural stem or progenitor cell
R: Regenerative
RNA-Seq: high-throughput RNA sequencing
ROS: reactive oxygen species
STRING: functional protein-protein interaction database
TCA: tricarboxylic acid cycle (Krebs cycle).
