# Supplementary material for: Quantitative Proteomics After Spinal Cord Injury (SCI) in a Regenerative and a Nonregenerative Stage in the Frog Xenopus laevis
Source: Mol Cell Proteomics. 2018 Jan 22;17(4):592–606. doi: 10.1074/mcp.RA117.000215 (PMC5880103; doi:10.1074/mcp.RA117.000215)
Supplement: Supplemental Data [file supp_17_4_592__index.html]

Quantitative Proteomics After Spinal Cord Injury (SCI) in a Regenerative and a Nonregenerative Stage in the Frog Xenopus laevis — Spinal Cord Regeneration Proteomics — Supplemental Data 

# Quantitative Proteomics After Spinal Cord Injury (SCI) in a Regenerative and a Nonregenerative Stage in the Frog *Xenopus laevis*

## Supplemental Data

- Supplementary data legends - Legends to all Supplementary data files
- Supplementary data 1 - Lists of quantified proteins, all differentially expressed proteins, and top 15 proteins with the highest fold-changes.
- Supplementary data 2 - Full gene ontology enrichment analysis results for regenerative and non-regenerative stages including protein names.
- Supplementary data 3 - Complete data of reporter ion intensities for all identified proteins and all quantified proteins before normalization, and after normalization for all quantified proteins.
- Supplementary data 4 - Full list of protein groups and peptide ID list.
- Supplementary data 5 - Reproducibility data for biological replicates (see file for individual figure legends).
- Supplementary data 6 - Full list of gene ontology (GO) categories that were enriched in both RNA-Seq and iTRAQ data.
