# Supplementary material for: Quantitative Proteomics After Spinal Cord Injury (SCI) in a Regenerative and a Nonregenerative Stage in the Frog Xenopus laevis
Source: Mol Cell Proteomics. 2018 Jan 22;17(4):592–606. doi: 10.1074/mcp.RA117.000215 (PMC5880103; doi:10.1074/mcp.RA117.000215)
Supplement: Supplemental Data [file supp_RA117.000215_4818_1_supp_46816_p1scml.pdf]

Supplementary data legends

Supplementary data 1. Lists of quantified proteins, all differentially expressed proteins, and top 15 proteins with the highest fold-changes.

Supplementary data 2. Full gene ontology enrichment analysis results for regenerative and non-regenerative stages including protein names.

Supplementary data 3. Complete data of reporter ion intensities for all identified proteins and all quantified proteins before normalization, and after normalization for all quantified proteins.

Supplementary data 4. Full list of protein groups and peptide ID list.

Supplementary data 5. Reproducibility data for biological replicates (see file for individual figure legends).

Supplementary data 6. Full list of gene ontology (GO) categories that were enriched in both RNA-Seq and iTRAQ data. Only level 5 GO terms are shown, and a separate sheet for each category is included to show the terms that were exclusively found in either the regenerative or the non-regenerative stage. Of note, no such terms were found for the cellular component category of the non-regenerative stage.
