# Supplementary material for: Quantitative Proteomics After Spinal Cord Injury (SCI) in a Regenerative and a Nonregenerative Stage in the Frog Xenopus laevis
Source: Mol Cell Proteomics. 2018 Jan 22;17(4):592–606. doi: 10.1074/mcp.RA117.000215 (PMC5880103; doi:10.1074/mcp.RA117.000215)
Supplement: Supplemental Data [file supp_RA117.000215_4818_1_supp_46821_p1scmm.pdf]

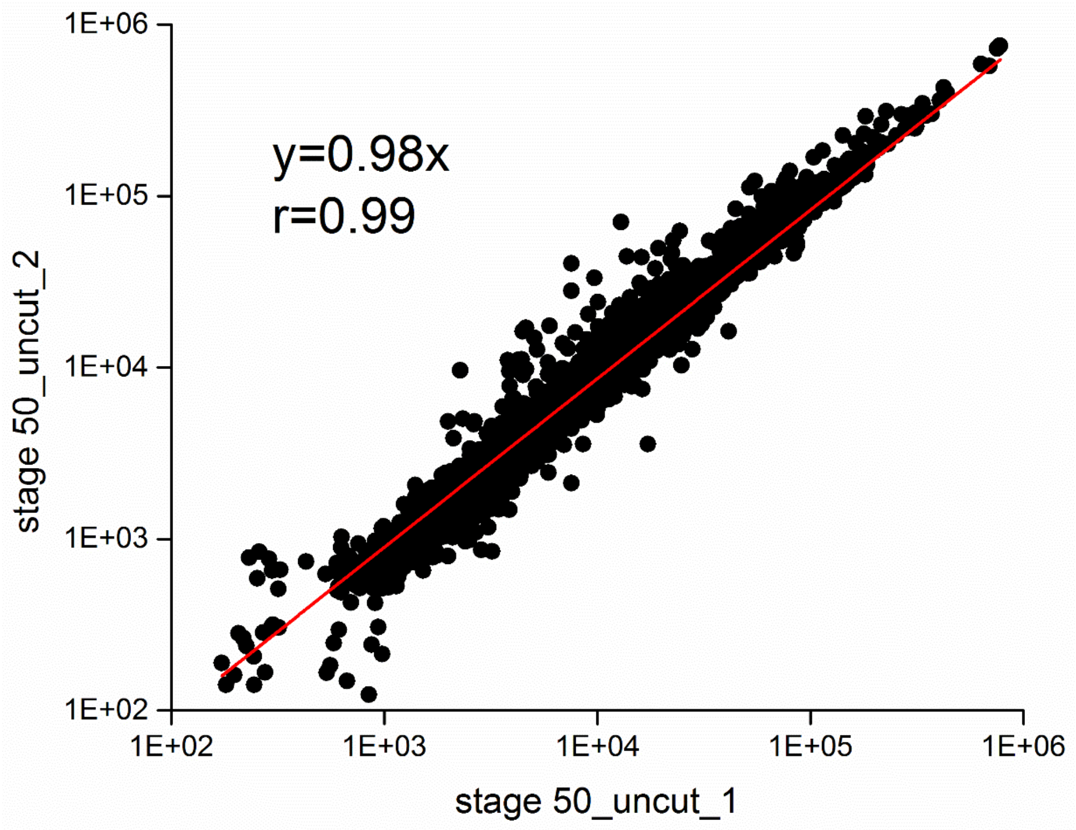

Figure S1: Correlation of reporter ion intensity between biological replicates of uncut samples at stage 50.

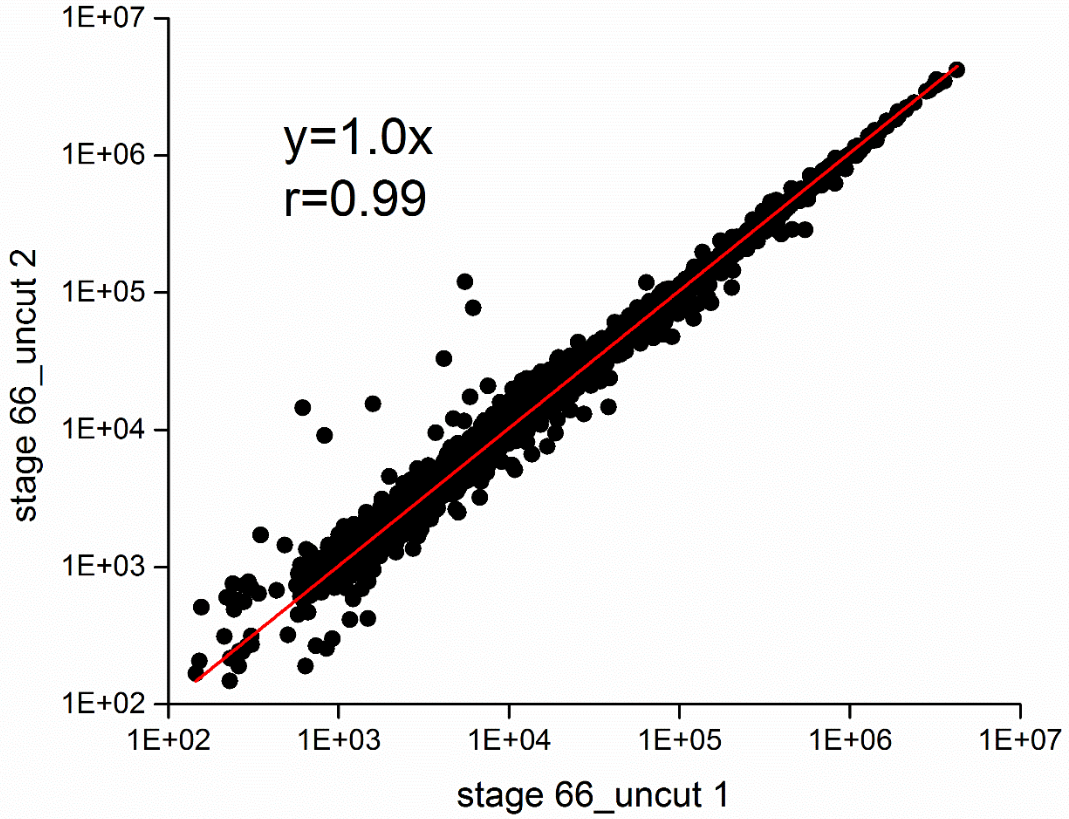

Figure S2: Correlation of reporter ion intensity between biological replicate of uncut samples at stage 66.

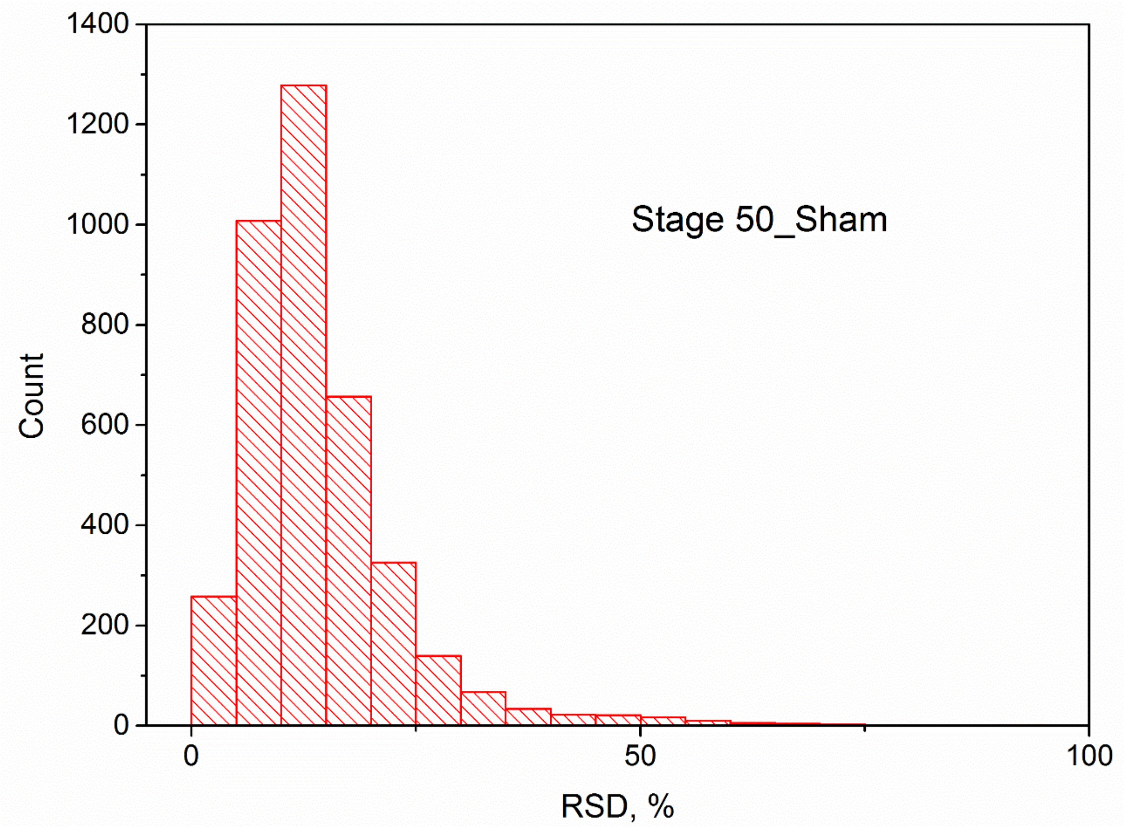

Figure S3. Distribution of the relative standard deviation (RSD) of reporter ion intensity from biological triplicate of sham samples at stage 50.

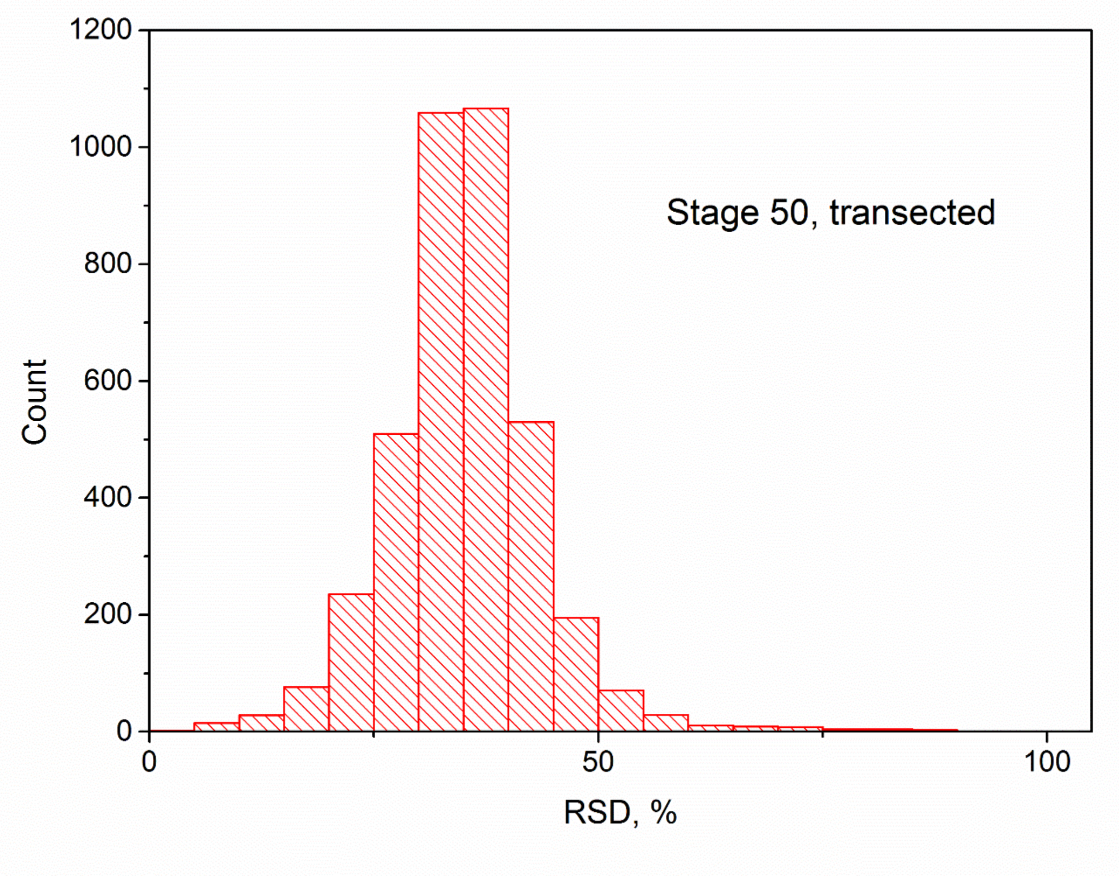

Figure S4. Distribution of the relative standard deviation (RSD) of reporter ion intensity from biological triplicate of transected samples at stage 50.

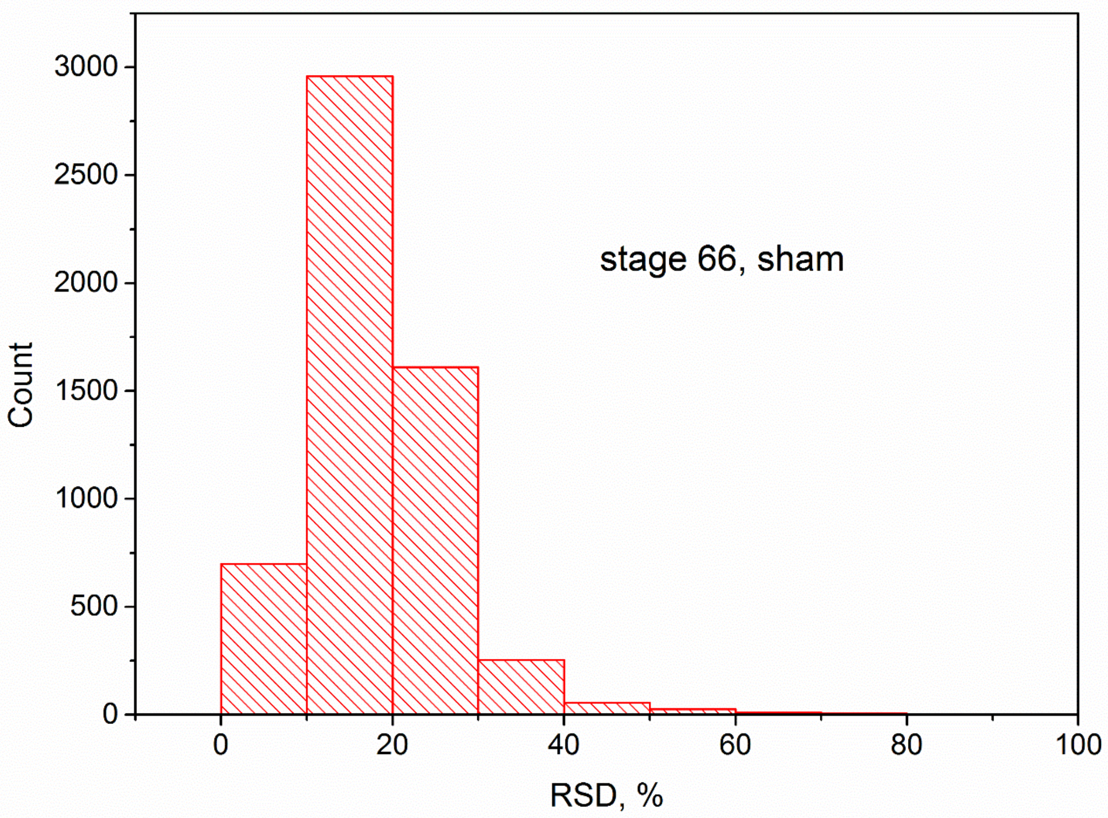

Figure S5. Distribution of the relative standard deviation (RSD) of reporter ion intensity from biological triplicate of sham samples at stage 66.

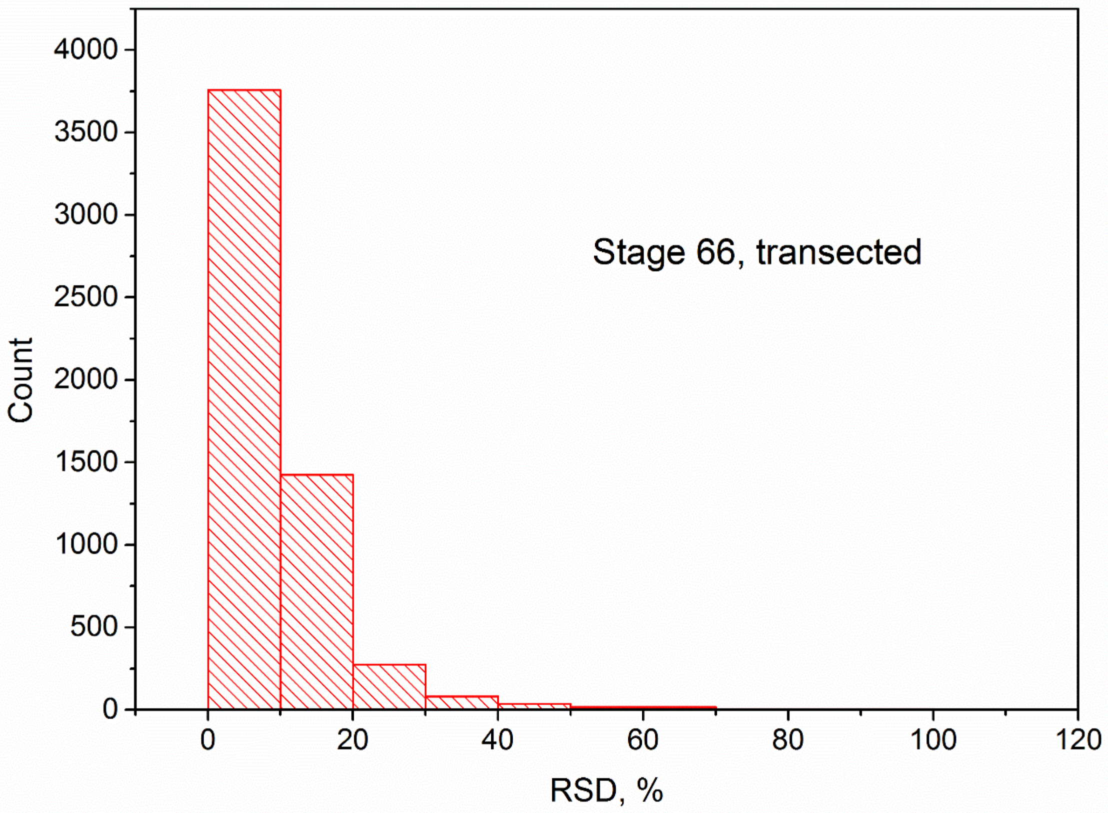

Figure S6. Distribution of the relative standard deviation (RSD) of reporter ion intensity from biological triplicate of transected samples at stage 66.
